# Early Implanon Discontinuation and Associated Factors among Implanon User Women in Debre Tabor Town, Public Health Facilities, Northwest Ethiopia, 2016

**DOI:** 10.1155/2018/3597487

**Published:** 2018-01-21

**Authors:** Mengstu Melkamu Asaye, Tewodros Syoum Nigussie, Worku Mequannt Ambaw

**Affiliations:** Midwifery Department, College of Medicine and Health Sciences, University of Gondar, P.O. Box 196, Gondar, Ethiopia

## Abstract

**Background:**

Implanon discontinuation closely related to higher rates of overall fertility rate, unwanted pregnancies, and induced abortion. This might have social and economic consequences. In Ethiopia the magnitude of early Implanon discontinuation and contributing factors is not well studied.

**Objective:**

To assess early Implanon discontinuation and associated factors among Implanon user women in Debre Tabor town, 2016.

**Methods:**

A facility based cross-sectional study was conducted from May 1 to August 2016 through face-to-face interview. A total of 449 Implanon user women were selected by systematic random sampling technique. Epi Info version 7 and SPSS version 20 were used for data entry and analysis, respectively. Factors associated with early Implanon discontinuation were analyzed using binary and multivariable logistic regression model. Variables with* p* value of <0.05 and 95% confidence interval were considered as statistically significant.

**Results:**

The overall proportion of early Implanon discontinuation among mothers was 65 % (95%, CI: 60.4%–69.5%). Having no children (AOR = 2.0, 95% CI = [1.3–4.5]), being not counseled for possible side effects (AOR = 1.50, 95% CI = [1.1–3.4]), having no appointment for follow-up (AOR = 2.6, 95% CI = [1.2–4.3]), and having developing side effects (AOR = 1.7, 95% CI = [1.5–4.4]) were found to be statistically significant factors associated with early Implanon discontinuation.

**Conclusion and Recommendation:**

Early Implanon discontinuation among mothers was found to be high. Hence, counseling about Implanon side effects and appointment for follow-up of Implanon users should be made to increase Implanon retention.

## 1. Introduction

### 1.1. Background

Contraceptive discontinuation is a worldwide problem that may be connected with low incentive to avoid pregnancy. Every year, about one-third of the 182 million pregnancies occurring worldwide are unplanned. Contraceptive discontinuation may indicate a missed opportunity to promote and sustain contraceptive use, and therefore it can be used to measure the effectiveness of family planning services [1].

Discontinuation of a contraceptive method often leads to unintended pregnancy, this leads to potentially unsafe induced abortions and unplanned births that expose a risk to the health of the women and ultimately reduced educational attainment [2].

The avoidance of intended pregnancies becomes less dependent on rates of initial adopted contraceptives and more dependent on the ability and willingness of couples to use methods with a maximum effectiveness [3].

In Ethiopia nearly 1 in every 4 married women (37%) of reproductive age group (15–49) does not want any more children. The vast majority of married women want a contraceptive method to either space their next birth or cease childbearing altogether. Among contraceptive users 8% and 11% of married and unmarried sexually active women are using implants [4]. In Amhara region 4% of married women are Implanon user [5] and in Debre Tabor town, the coverage of Implanon utilization is 8.2% [6].

In general, when couples discontinue using a contraceptive method, even for a brief period, the women run the risk of becoming pregnant unintentionally. Those unintended pregnancies often lead to larger than intended family sizes and contribute to higher rates of overall fertility [7].

### 1.2. Statement of the Problem

Early Implanon discontinuation is defined as discontinuation at less than 2.5 years after insertion of Implanon [8, 9].

In a cross-sectional study in Zaria Nigeria, early Implanon discontinuation was 19% and 69% of removal reason was menstrual disruption [10]. In a community-based cross-sectional study at Ofla Tigray, early Implanon discontinuation was 16% with mean (±SD) of 6.6 ± 2.8 in months [11].

Implanon is an effective form of family planning. Contraceptive discontinuation is a worldwide incident and highly contributes substantially to unplanned pregnancies, unwanted births, and termination of pregnancies [1, 10].

In sub-Saharan Africa including Ethiopia, there are high population and reproductive health challenges, which are indicating higher maternal mortality, higher total fertility rate, and unintended pregnancies [6].

When a woman discontinues the prevailing family planning methods in her body, she may become pregnant unintentionally. This unplanned pregnancy has impacts on larger family size and ultimately contributes to higher overall fertility rates and reflects the effectiveness of family planning program [12], which may have social and economic consequences [3].

When emphasis is given on possible side effect counseling, reassurance, and follow-up, duration of Implanon utilization and women's satisfaction would be increased. Implanon discontinuation rates are poorly documented in Africa [5].

Apart from low utilization, early Implanon discontinuation and its determinants among Implanon user women in Ethiopia are not well studied. The purpose of this study was to assess early Implanon discontinuation and identify its associated factors among Implanon users at Debre Tabor town.

### 1.3. Literature Review

#### 1.3.1. Proportion of Early Implanon Discontinuation

Contraception and fertility planning should form part of every consultation. Contraception is a means for reducing maternal mortality and morbidity associated with unplanned pregnancy [2] and also prevents pregnancy in women who are medically unfit for pregnancy until their condition has been improved [3].

In a community-based cross-sectional study, United States (US), early Implanon discontinuation was 25.2% with mean duration of utilization 10.4 months and 15% of removal reason was menstrual disruption [14].

In a cohort study conducted in Netherland, discontinuation rate of Implanon after 12 months was 28% but it was 47% at 24 months. History of previously used contraceptive method was associated with highest continuation rates and menstrual disruption was the main reason of early Implanon discontinuation [12].

In a retrospective study at Australia, at 6 months after insertion, 6% of women had removed Implanon. The discontinuation rates showed that 26% removed at one year, 39% discontinued at one and a half years, and 50% removed at two years. Around 40% of women were still using Implanon more than 2.5 years [15].

On the other hand, in a retrospective study in Jordan, many women discontinue contraceptive use within one year of initiating a method. The one-year probability of discontinuing overall contraceptive was 45% [7].

In a retrospective study conducted in low income certain African countries, the discontinuation rates of implants were as follows: Egypt 36%, Yemen 43%, and Cambodia 45% before 2 years after insertion with different reasons [2]. It leads to high prevalence of unintended pregnancy due to its early implant discontinuation.

In a descriptive retrospective study, Ilorin, Nigeria, early Implanon discontinuation rate was 26.5% [16] and on the other hand, in a community-based cross-sectional study at Ofla Tigray, early Implanon discontinuation rates were 16% [11].

#### 1.3.2. Factors Associated with Early Implanon Discontinuation

Discontinuation of contraceptive occurs, when a woman has no need for longer utilization or a woman has not counseled about side effects of the method [2].

In a cross-sectional retrospective study conducted in Port, Nigeria, postinsertion follow-up and counseling of side effects will increase continuation of Implanon utilization and retention [17].

In a retrospective cross-sectional study, Jos, Nigeria, the indication of early Implanon removal was 33.3% due to menstrual disruption. The other indication for early Implanon discontinuation was desire for another pregnancy closely followed by 30% [18], and in another cohort study, unmarried women had 1.62 odds to discontinue compared to married women and younger (14–19 years) and were also associated with early discontinuation [19].

In Columbus, Ohio, discontinuation due to bleeding abnormalities was 77.5% and wanting a baby was the other reason for early Implanon removal. Through counseling of a method prior to placement specially about abnormal bleeding, it increases utilization and retention of Implanon [20].

In a study conducted in Jordan, to increase continuation of a contraceptive method, male involvement counseling was very advantageous. Counseling about possible side effects with counseling material increases 1.6 times of client satisfaction which also increases duration of utilization of a family planning method [21] and in another study on this country, multivariate logistic regression of side effects like menstrual disruption was associated with early removal of implants (*p* = 0.002) and preplacement counseling regarding significant side effects may have an impact on early removal [9].

In a cohort study in Ankara, Turkey, early Implanon discontinuation was 39% due to developing side effects. 18% of the main removal reasons were due to menstrual disruption and 5% due to weight gain [22]. On the other hand, in a cohort study in Philippines, quality of care was correlated for continuation of a contraceptive method. The rate of discontinuation was higher among women who reported that they had not been counseled about side effects of the method [23] and in a study conducted in India, 29% early Implanon discontinuation was due to 30.5% menstrual disruption [24].

In institution based cross-sectional study, in Bangladesh, younger women had higher rate of contraceptive discontinuation of any method, especially those with no children. Women with 1 or 2 living children had 0.49 odds compared to those with no children for contraceptive discontinuation. Women who did not intend to have other children were less likely to discontinue any contraceptive [1].

In a descriptive retrospective study, Ilorin, Nigeria, indications for early discontinuation were as follows: 35% was due to desire of pregnancy followed by 25% being due to menstrual disruption [16].

In Jos Nigeria, mean of 13.4 ± 6.8 months was the duration of Implanon utilization. The suggested removal reasons were 53.1% side effect of the method. From side effects 33.3% was menstrual disruption followed by 13.3% being due to weight gain [18].

In a institution based cross-sectional study in Arsi, Oromia region, 25% of early Implanon discontinuation was mainly due to menstrual disruption whereas 24% was due to plan to conceive in the near future with the mean duration of 19.5 months [13].

On the other hand, in a community base cross-sectional study at Ofla Tigray, the discontinuation rate of Implanon was 16% with different factors. Among the associated factors, women who developed side effects were 2.8 odds, women who were not appointed for follow up and women who were not satisfied by the service given during the insertion of Implanon were more than 3 times more likely to discontinue Implanon as compared to their counterparts [11].

### 1.4. Conceptual Framework

 See Figure 1.

### 1.5. Justification of the Study

Contraceptive implants are the most effective family planning methods available and well-accepted worldwide. Despite its proven long protection, only 8% of married women in Ethiopia use implants and 8.2% of all reproductive age women use Implanon in the study area [4, 6]. Apart from its low utilization, premature removal is common with unknown reasons. Contraceptive discontinuation highly contributes to unplanned pregnancy, abortions, and maternal deaths.

To date there was no study conducted on early Implanon discontinuation and associated factors among Implanon user women in the study area. Therefore, this study aimed to assess early Implanon discontinuation and determinant factors among Implanon user women.

The findings of this study will in general help the health professionals to understand the extent of early Implanon discontinuation. In addition to this, the finding will enhance the capacity of planning and decision making to look for possible solutions to solve the problem in collaboration with concerned stake holders.

Moreover, the findings will help policy makers and planners and other concerning organizations working in the area of family planning and maternal health to plan new strategies based on the identified factors to improve early discontinuation of Implanon in the community.

Finally the findings from this study will help to achieve sustainable development goal 3 (SDGS) of family planning in the country.

## 2. Objectives

### 2.1. General Objective

- To assess early Implanon discontinuation and associated factors among Implanon user women in Debre Tabor town health institutions, South Gondar Zone, Amhara Region, northwest of Ethiopia, from May 1 to August 30, 2016.

### 2.2. Specific Objectives

- To determine proportion of early Implanon discontinuation among Implanon user women in Debre Tabor town health institutions.
- To identify factors associated with early Implanon discontinuation among Implanon user in Debre Tabor town health institutions.

## 3. Methods

### 3.1. Study Design and Period

Facility based cross-sectional study was conducted at Debre Tabor town public health facilities from May 1 to August 30, 2016.

### 3.2. Study Area

Debre Tabor town is found in South Gondar Zone of Amhara regional state of Ethiopia. It is located 98 kilometer far from Bair Dar, the city of Amhara regional state, and 666 kilometer far from Addis Ababa, the capital city of Ethiopia, to the northwest of the country. The town has four administrative kebeles with a total population of 67,485; among these 13,969 were in the reproductive age group and 96.72% of the population of the town are Orthodox Christians whereas 2.54% is Muslim [25]. There are one general hospital, 4 health centers, and two medium private clinics in the town.

### 3.3. Source and Study Population

#### 3.3.1. Source Population

 The study includes all women of reproductive age group (15–49 years of age) who were using Implanon.

#### 3.3.2. Study Population

 The study includes all women who requested removal of Implanon following insertion during the study period at the study areas for any reason.

#### 3.3.3. Inclusion Criteria

 The study includes all women who requested removal of Implanon during the study period.

### 3.4. Sample Size Determination

In this study, sample size was determined using Open Epi version 2 by considering the following statistical assumptions. The odds ratio and counseling status about effectiveness was taken from previous study in Tigray Ethiopia [11].

*Assumptions*

Two sided significant level (1 − alpha): 95%.
Power (1 − beta, % chance of detecting): 80.
Ratio of sample size: 1.
Percent of not counseled about effectiveness with outcome: 9.6.
Percent of counseled about effectiveness with outcome: 20.
Odds ratio: 2.36.
Continuity correction result = 404 which is the largest sample size from the factors.
Then by adding 10% none response rate
  (1) $$\begin{array}{c} n=404*\frac{1}{1-0.1}=449. \end{array}$$

### 3.5. Sampling Procedure

**Figure 2:**
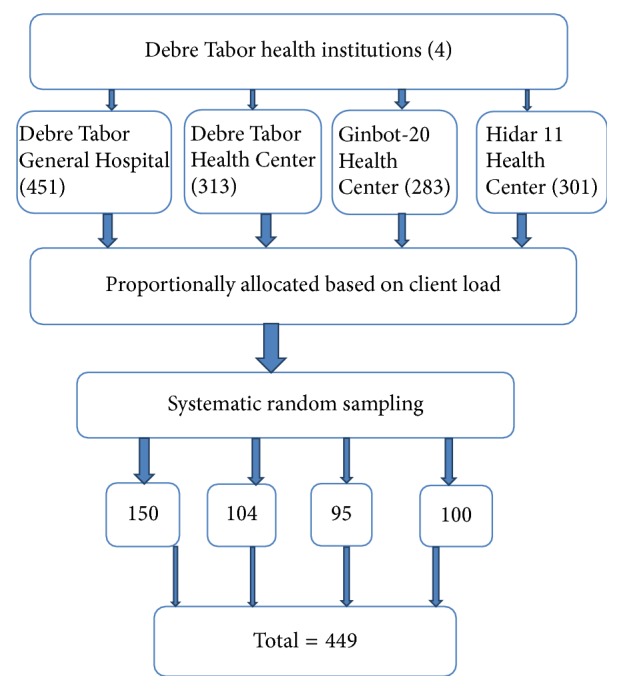
Schematic presentation of sampling procedure for early Implanon discontinuation and associated factors in Debre Tabor town public health facilities, South Gondar Zone, Amhara Region, 2016.

 Public health facilities which gave the service were Debre Tabor Hospital, Debre Tabor-Health Center, Ginbot-20 Health Center, and Hidar 11 Health Center and all were included in the study. The calculated sample size was proportionally allocated to each health facility based on the previous consecutive five month average daily client flow of the units which were obtained by referring client registration log books. The average five-month client flow for Debre Tabor Hospital, Debre Tabor-Health Center, Ginbot-20 Health Center, and Hidar 11 Health Center was 451,313,283 and 301, respectively. A total of 1347 women were booked for Implanon removal in five months from review of the previous months' record of each health institutions. The study participants were selected by using systematic random sampling method from Implanon removal women who visited the health institutions during the data collection period. The first client in each health facility was selected by lottery method. We compute *K*th for each health facility and it ranges from 2.66 to 2.78; then every 3rd Implanon user woman in each health facility from their sequence of family planning visit in the study period was selected (Figure 2).

### 3.6. Study Variables

#### 3.6.1. Dependent Variable

- Early Implanon discontinuation

#### 3.6.2. Independent Variables

- Sociodemographic character: age, marital status, religion, occupation, and education.
- Obstetric factors: number of children, parity, and abortion.
- Social factors: husband objection, husband involvement, and husband go abroad and neighbors influence.
- Method related factors: side effects, past contraceptive utilization, desire for pregnancy, follow-up, counseling, and weight gain.

### 3.7. Operational Definitions

- Contraceptive: an agent or device intended to prevent conception.
- Counseling: making the women aware of its long protection, side effects, and effectiveness of the method.
- Early Implanon discontinuation: removal of Implanon by health professionals before 2.5 years of utilization.
- Long acting reversible contraceptive: contraceptive methods which serve as 3–10 years but can be removed at any time.
- Menstrual disruption: any deviation of a women's regular menstrual cycle.
- Unintended pregnancy: pregnancy without mother's plan.
- Side effect: when the women develop at least one side effect after Implanon insertion like menstrual disruption, insertion arm pain, headache, acne, and others.

### 3.8. Data Collection Tools

Data were collected using semistructured face-to-face interviewed questionnaire having three parts. The first part contains sociodemographic characteristics of mothers. The second part of the questionnaire was obstetric characteristics of women and the third part was contraceptive related characteristics. One midwife supervisor and four 2nd-year diploma midwifery students were employed for data collection and trained for half of a day about the purpose of the study, timely collection of data, and overall data collection procedure.

### 3.9. Data Quality Control

To assure the quality of the data the questionnaire was pretested 1 week before the actual data collection time on 23 Implanon discontinuer women at Adiszemen Hospital and appropriate modification was made. The questionnaire was prepared in English and translated into Amharic and retranslated back to English by two language experts. Data collectors and supervisor were trained for half day before one week of the actual data collection time. Interviewers were supervised at each site and regular meetings had been held between the data collectors and the supervisor. The collected data were reviewed and checked for completeness before data entry. Data entry format template was produced and the data were entered into Epi Info version 7 to control data entry errors.

### 3.10. Data Processing and Analysis

Before analysis data clean-up and cross-checking were done. Data were checked, coded, and entered to Epi Info version 7 then it was exported to SPSS version 20 for analysis. Both descriptive and analytical statistical procedures were utilized. Descriptive statistics like percentage, mean, median, and standard deviation were used for the presentation of sociodemographic data and prevalence of early discontinuation of Implanon. Tables were also used for data presentation. Binary logistic regression was used to identify factors associated with early discontinuation of Implanon. Variables with *p* value less than 0.2 in bivariate analysis were entered into multivariable logistic regression model. Multiple logistic regression models was fitted to control the possible effect of confounders and finally the variables which had independent association with early discontinuation of Implanon were identified on the basis of OR, with 95% CI and *p* value less than 0.05. The variables were entered in the multivariate model using the Backward Stepwise regression method. Model fitness was checked using Hosmer and Lemeshow goodness of fit test (*p* = 0.65).

## 4. Ethical Clearance

Ethical approval and clearance were obtained from Institutional Review Board of University of Gondar. Support letter was obtained from University of Gondar to Amhara Regional Health Bureau and South Gondar Zone Health Office. Letter of cooperation was secured from the administrations of each health institution. Informed verbal consent was obtained from study participants to confirm willingness to participate after explaining the objectives, benefits, and risks of the study. Participation in the study was voluntary and a study participant has the right to accept or refuse participation in the study at any time.

Confidentiality was assured and no personal details were recorded in any documentation related to this study.

## 5. Result

### 5.1. Sociodemographic Characteristics

A total of 449 women have responded to the questionnaires making a response rate of 100%. The age of the study participants was between 16 and 45 years with mean (±SD) age 26.7 ± 6.7 years. More than half, 332 (73.5%), of the respondents were married, 394 (87.8%) were Orthodox Christians, 327 (73%) were urban in residence, and all were Amhara in ethnicity.

Among the participants 195 (43.4%) were house wives and 136 (41%) of their husband were employed by occupation and 166 (37%) of the participants and 161 (48.5%) of their husbands had educational status of college and above (Table 1).

### 5.2. Obstetrics Related Characteristics

Obstetrics history was one of the factors that were assessed in the study. One hundred sixty-nine (37.7%) respondents had given birth one to two times while 154 (34.3%) of the study women were nulliparous. Out of those who had gave birth 95 (21.2%) women had one to two alive children and 47 (10.5%) had more than five alive children.

Among 449 respondents, 101 (22.5%) of them had abortion history and 332 (73.9%) study participants had desire to have more children in the near future. From the participants who desire to have child 154 (46.4%) of them need to have child within two years (Table 2).

### 5.3. Contraceptive and Counseling Related Characteristics

Majority of the participants 374 (83.3%) used modern contraceptive before the currently discontinued Implanon. Out of them 221 (59.1%) were using injectable followed by oral contraceptive pills 88 (23.5%).

Two hundred eighty (62.4%) and one hundred three (31.8) of the participants got counseling service about benefit and side effect of Implanon during insertion, respectively. All of study participants got Implanon insertion at government health institutions and 269 (70%) of them choose Implanon by their own.

Two hundred ten (46.8%) of study participants did not have appointment follow-up during their Implanon utilization period.

The suggested reasons for premature removal of Implanon before due date were identified that side effect of Implanon was the major, which accounted for 264 (71%) followed by 61 (16.4%) of plan to conceive in the near future (Table 3).

**Figure 3:**
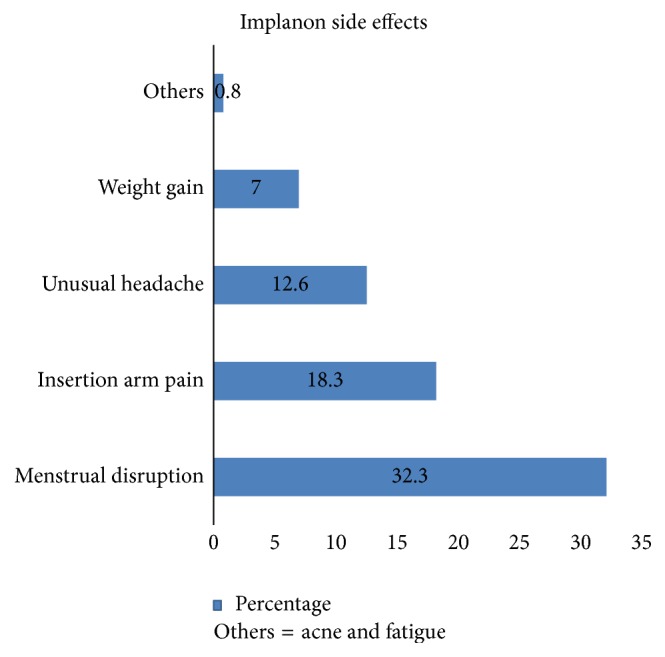
The main side effects of Implanon for early discontinuation among Implanon user women in Debre Tabor town public health facilities, South Gondar Zone, Amhara Region, 2016.

Among women who developed side effects, menstrual disruption accounted 120 (32.3%) for early Implanon discontinuation followed by 68 (18.3%) insertion arm pain (Figure 3). Proportion of Early Implanon Discontinuation

**Figure 4:**
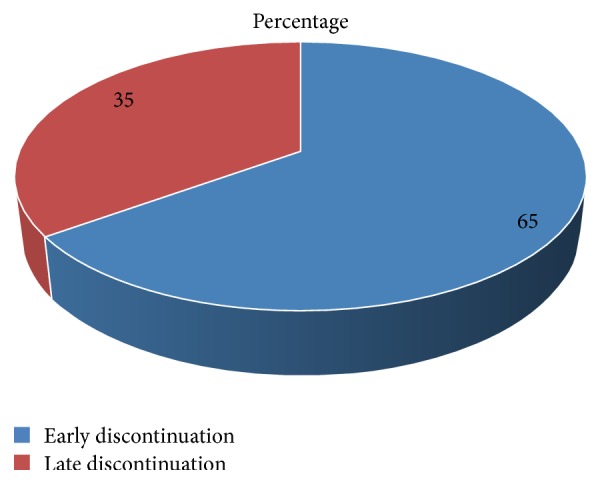
Proportion of early Implanon discontinuation among Implanon user women in Debre Tabor town public health facilities, South Gondar Zone, Amhara Region, 2016.

The main concern of this study was to assess the proportion of early Implanon discontinuation among women who ever used it and request its removal. From 449 study participants who had Implanon removal, 292 (65%, 95% CI, 60.4%–69.5%) were overall early discontinuation. The mean (±SD) overall duration of Implanon utilization in months was 21.5 ± 8.4 (Figure 4).

### 5.4. Factors Associated with Early Implanon Discontinuation

Finding from bivariate logistic regression analysis revealed that age, residence, parity, number of living children, plan to conceive in the near future, counseling about possible side effects, appointment for follow-up, and developing side effects had associated with early discontinuation of Implanon. However, in multivariable logistic analysis only, women with no living children, being not counseled for possible side effects, having developing side effects, and having no appointment for follow-up given during Implanon insertion found significant associated factors with early Implanon discontinuation.

According to this study, the odds of discontinue Implanon early among women with no living children were 2 times than those who had living children (AOR = 2.0; 95% CI: 1.3–4.5).

The study suggested that the odds of discontinuing Implanon early among women who did not counsel about possible side effects were 1.5 times than those who got counseling service about Implanon side effects (AOR = 1.5; 95% C.I: 1.1–3.4).

The analysis showed that the odds of discontinue Implanon early among women who did not appointed for follow-up were 2.6 times than those who had appointment follow-up (AOR = 2.6; 95% C.I: 1.2–4.3).

The study also revealed that the odds of discontinue Implanon early among women who develop side effects were 1.7 times than those who had requested premature removal due to none side effects (AOR = 1.7; 95% C.I: 1.5–4.6) (Table 4).

## 6. Discussion

This study assessed the proportion of early Implanon discontinuation among women who request removal.

The proportion of early Implanon discontinuation among women who ever used Implanon was 65% with mean duration of 21.5 ± 8.4 months. This finding is in agreement with study conducted at Australia, 60% [15]. However, the current proportion is higher as compared to studies conducted in Netherland, 47% [12], Yemen, 43%, and Cambodia, 45% [2], US, 25.2% [14], India, 37% [24], Zaria, 19% [10], and Ilorin Nigeria, 26.5% [16]. The difference could be attributed to lack of preinsertion counseling particularly about the possible side effects of the method as compared to other studies [7, 20]. In addition to this, it might be due to the educational status of the study participant's as nearly half of the current studies were below primary level as compared to other studies [2, 10, 12, 14, 16, 24]. Moreover, it might be due to lack of efforts made to improve counseling, specially, on those mothers having menstrual disruption problems. This might have an impact on high proportion of early Implanon discontinuation. Lastly, it might be due to sociocultural differences of respondents across the study areas.

Women with no living children, who did not counsel about side effects of Implanon, who had no appointment for follow-up, and those who developed side effects significantly associated with early Implanon discontinuation.

According to this study, the odds of discontinues Implanon early among women with no living children were 2 times than those who had living children. This is in line with study conducted in Bangladesh [1] and Burkina Faso [2]. This is related to the fact that women with no living children might intend to have children and due to the fact that they may have higher discontinuation proportion. In addition to this, 35% of the present study women were between 22 and 27 years of age. Since they were young, they may intend to have more children and discontinue Implanon early [19].

In the present study, the odds of discontinue Implanon early among women who did not counsel about possible side effects were 1.5 times than those who got counseling service of Implanon side effects. This is consistent with study conducted in Tigray [11], Philippine [23], and Jordan [21]. Providing counseling during insertion of Implanon was positively associated with continuation of use [3, 23]. Preplacement counseling about the possible side effects of the method [5] and support by the service providers might be the most important way to help women continue on Implanon contraception. Women and couples who receive better counseling on their method may be more aware of potential side effects and how to cope with them. Preinsertion counseling on Implanon side effects is indispensable to increase the duration of Implanon being used [13]. In addition to this, preplacement counseling regarding significant side effects, including male involvement, may have an impact on early Implanon discontinuation.

The analysis also showed that the odds of discontinue Implanon early among women who did not had appointment for follow-up were 2.6 times than those who had appointment for follow-up. This is in agreement with study conducted in Tigray [11]. This might be because of feeling of confidence due to caring professionals and treating of possible side effects early.

This study also suggested that the odds of discontinue Implanon early among respondents who developed side effects were 1.7 times than those who had requested premature removal due to none side effects. This is consistent with study conducted in turkey [22], Tigray [11], Jos Nigeria [18], and Jordan [9]. It might be because experiencing side effect of the method could contribute for early Implanon discontinuation. In addition to this, respondents who had discontinued Implanon due to side effects might be because of intolerance of the side effects and fear of different complications to occur may lead to discontinuation of the method early. Moreover, this might be because of respondents who might be more concerned about culturally sensitive issues like vaginal bleeding and would want the Implanon to be removed early in order not to interfere with their sexual relationship with their husband.

## 7. Limitation of the Study

As the study was institutional based and conducted only on Implanon user women who request removal, it might not be generalized to the majority general population including the rural community. Since the study design was cross sectional; therefore, it may be difficult to establish temporal relationship.

## 8. Conclusion

Proportion of early Implanon discontinuation in this study was found to be high. Factors like women with no living children, mothers who did not receive counseling fort side effects of Implanon, women who had no appointment for follow-up, and those mothers who developed side effects were found statistically associated with early Implanon discontinuation.

## 9. Recommendation

*To South Gondar Health Office and Other Stake Holders. *Government health organizations and other stake holders could develop programs to work on health care providers in order to increase retention of Implanon utilization.

*To Health Service Providers. *Health professionals could be give preinsertion counseling with giving emphasis on possible Implanon side effects.

Follow-up of Implanon users could be made by health professionals to increase retention.

Early side effect management and reassurance is recommended to decrease early discontinuation.

*To Researchers. *Further comprehensive study including health professionals and a community-based study is recommended.

## Figures and Tables

**Figure 1:**
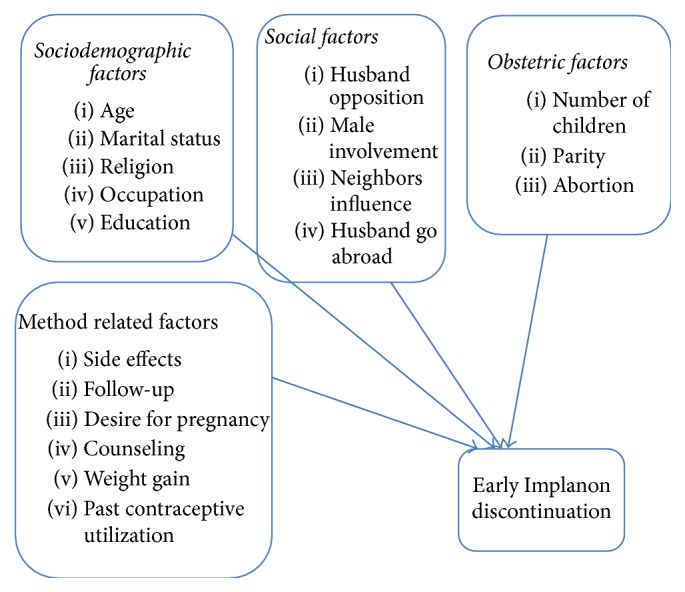
Conceptual framework adapted from review of literatures on early Implanon discontinuation and associated factors [11, 13].

**Table 1:** Sociodemographic characteristics of Implanon users in Debre Tabor town public health facilities, northwest Ethiopia, August 2016 (*n* = 449).

Variables	Number	Percentage
Women's age		
16–21	115	25.6
22–27	157	35
28–33	107	23.8
34–39	35	7.8
40–45	35	7.8
Marital status		
Married	332	73.9
Single	71	15.8
Divorced	34	7.6
Widowed	12	2.7
Religion		
Orthodox	394	87.8
Muslim	34	7.6
Others^*∗*^	21	4.6
Residence		
Urban	327	73
Rural	122	27
Women's education		
Illiterate	93	20.7
Read and write	33	7.3
Primary	81	18
Secondary	76	16.9
College and above	166	37
Husband's education (*n* = 332)		
Illiterate	54	16.26
Read and write	39	11.75
Primary	34	10.24
Secondary	44	13.25
College and above	161	48.49
Women's occupation		
House wife	195	43.4
Employee	120	26.73
Student	87	19.38
Trader	47	10.47
Husband's occupation		
Employee	136	41
Trader	59	18
Student	20	6
Farmer	117	35

*∗* = catholic and protestant.

**Table 2:** Obstetrics characteristics of Implanon user women in Debre Tabor town public health facilities, northwest Ethiopia, August 2016 (*n* = 449).

Variables	Number	Percentage
Parity		
0	154	34.3
1-2	169	37.7
3+	126	28
Living children		
0	156	35
1-2	95	21.2
3-4	91	20.3
5+	47	10.5
History of abortion		
Yes	101	22.5
No	348	77.5
Desire for pregnancy		
Yes	332	73.9
No	117	26.1
When they want become pregnant (*n* = 332)		
Within two years	154	46.4
After two years	178	53.6

**Table 3:** Contraceptive related characteristics of study participants in Debre Tabor town public health facilities, northwest Ethiopia, August 2016 (*n* = 449).

Variables	Number	Percentage
Ever used contraceptive before Implanon		
Yes	374	83.3
No	75	16.7
Type of contraceptive used before Implanon		
OCP	88	23.5
Injectable	221	59.1
OCP and injectable	60	16
Others^*∗*^	5	1.4
Place of insertion of Implanon		
Hospital	119	26.5
Health center	271	60.4
Health post	59	13.1
Counseling for benefit of Implanon		
Yes	280	62.4
No	169	37.6
Who choose Implanon		
Own choice	269	59.9
Health professional	130	29
Health extension	27	6
Others^*∗∗*^	23	5
Counseling about Implanon side effects		
Yes	143	31.8
No	306	68.2
Follow-up		
Yes	209	46.5
No	240	53.5
Removal due to side effect		
Yes	264	71
No	108	29
Nonside effect removal reasons		
Desire for pregnancy	61	16.4
Husband objection	34	9.2
Divorce	8	2
Husband go abroad	5	1.4

*∗* = IUCD, Jadelle, and Implanon; *∗∗* = husband and neighbor.

**Table 4:** Factors associated with early Implanon discontinuation among Implanon user women in Debre Tabor town public health facilities, northwest Ethiopia, 2016.

Variables	Early Implanon discontinuation		COR (95% C.I)	AOR (95% C.I)
	Yes (%)	No (%)		
Age				
16–34	271 (68.8%)	123 (31.2%)	1.6 (1.780–2.727)	- - - - - - - -
≥35	32 (58.2%)	23 (41.8%)	1	
Residence				
Urban	215 (65.5%)	112 (34.3%)	1	
Rural	88 (72.1%)	34 (27.9%)	1.3 (0.854–2.129)	- - - - - - - -
Parity				
≤1	175 (74.5%)	61 (25.8%)	1.9 (1.277–2.843)	- - - - - - - -
≥2	128 (60.1%)	85 (39.9%)	1	
Living children				
No children	116 (74.3%)	40 (25.7%)	2.3 (1.533–2.495)	2.0 (1.29–4.541)^*∗∗*^
Have children	145 (62.2%)	88 (37.8%)	1	
Desire of pregnancy				
Yes	230 (69%)	102 (31%)	1.5 (0.831–1.998)	- - - - - - - -
No	70 (60%)	47 (40%)	1	
Side effect counseling				
Yes	80 (60%)	63 (40%)	1	
No	222 (72.5%)	84 (27.5%)	2.1 (1.416–2.561)	1.5 (1.104–3.427)^*∗*^
Appointment for follow up				
Yes	124 (59.3%)	85 (40.7%)	1	
No	185 (77%)	55 (23)	2.4 (1.897–2.979)	2.6 (1.248–4.305)^*∗∗*^
Removal due to side effect				
Yes	200 (75.6%)	64 (24.4%)	2.0 (2.010–3.244)	1.7 (1.452–4.604)^*∗*^
No	65 (60.2%)	43 (39.8%)	1	

*Note*. *∗* = statistically significant at *p* < 0.05 and *∗∗* = statistically significant at *p* < 0.001.

## Abbreviations

AOR: Adjusted Odds Ratio
CSA: Central Statistical Agency
EDHS: Ethiopian Demographic and Health Survey
HEW: Health Extension Worker
IUCD: Intrauterine Contraceptive Device
LARC: Long Acting Reversible Contraceptives
OCPs: Oral contraceptive pills
SDGS: Sustainable Development Goals
SPSS: Statistical Package for Social Science
US: United States
UOG: University of Gondar.

## Supplementary Materials

Supplementary Materials Annex I: information sheet. Annex II: participants consent form. Annex III: English version questionnaire. Annex V: assurance of the investigator.
