# Supplementary material for: Early Implanon Discontinuation and Associated Factors among Implanon User Women in Debre Tabor Town, Public Health Facilities, Northwest Ethiopia, 2016
Source: Int J Reprod Med. 2018 Jan 21;2018:3597487. doi: 10.1155/2018/3597487 (PMC5896376; doi:10.1155/2018/3597487)
Supplement: Supplementary Materials — Annex I: information sheet. Annex II: participants consent form. Annex III: English version questionnaire. Annex V: assurance of the investigator. [file 3597487.f1.docx]

11. Annexes

Date: ------------Questionnaire code number: -----------Name of health facility: -----

Annex I: Information sheet

**Study Title:** Early Implanon discontinuation and associated factors among Implanon user women at Debre-tabor town public health facilities.

**Information sheet**

Hello, my name is ___________________________I am here today to collect data on behalf of Mengstu Melkamu study at University of Gondar. He is conducting a research for the partial fulfillment of second degree on early Implanon discontinuation and associated factors. You are selected to be one of the participants in this study and you will be kindly requested to answer for questionnaire including socio-demography, obstetric, past and current contraception history. In your participation in this assessment is voluntarily and your response will remain confidential. We hope you will be agree to participate as your views on the questions of this research are important. The interview may last about 25 minutes. Do you have any questions about this assessment?

Are you willing to participate in this study?

Yes--------------------------------------------------continue the next page

No --------------------------------------------------skip to the next participant

Annex II: Participants consent form

**Study Title:** Early Implanon discontinuation and associated factors among implanon user women at debre-tabor town public health facilities.

I have read/listen the participant information sheet and understand the following.

1. That I am free to withdraw at any time-----------------------------
2. That all information I provide will be dealt with in a confidential manner---------
3. I agree the researcher may contact me-------------

Signed. ----------------------Address --------------------------telephone No-------------

Annex III: English version Questionnaire

1. English questionnaire for assessment of early Implanon discontinuation and associated factors among implanon user women at debre-tabor town public health facilities.
   1. **Socio-demographic information**

| NO | Questions | Alternatives | Skip |
| --- | --- | --- | --- |
| 101 | Age | ----------years  I don’t know--------------  Refuse to tell------------ |  |
| 102 | Marital status | Single----------------------------1  Married--------------------------2  Divorced------------------------3  Widowed------------------------4 |  |
| 103 | Religion | Orthodox ---------------------1  Muslim-------------------------2  Protestant---------------------3  Catholic------------------------4  Other (specify)---------------5 |  |
| 104 | Residence | Urban---------------------------3  Rural ---------------------------2 |  |
| 105 | Ethnicity | Amhara-------------------------1  Tigrie----------------------------2  Oromo --------------------------3  SNNP ---------------------------4  Other(specify)------------------5 |  |
| 106 | Occupation | Government employee -------1  House wife------------------------2  Non-government employee --3 Merchant --------------------------4  Farmer ---------------------- ------5  Student ---------- ----------------6  other (specify) --------------------7 |  |
| 107 | Maternal educational level | Illiterate ---------------------1  Read and write-------------------2  Elementary -----------------------3  Secondary-------------------------4  College and above-------------- 5 |  |
| 108 | Husband ‘s educational level | Illiterate ---- ----------------1  Read and write-------------------2  Elementary -----------------------3  Secondary-------------------------4  College and above--------------5 |  |
| 109 | Husband’s occupation | Government employee---------1  Non-government employ------ 2 Merchant ------- --------3  Farmer --------------------------- -4  Student ---------------------------5  other (specify)--------------------6 |  |

**1.2 Obstetric related issues**

| **No** | **Questions** | **Alternatives** | **skip** |
| --- | --- | --- | --- |
| 201 | Parity | ---------------------------------------------------- |  |
| 202 | Number living children | ----------------------------------------------------- |  |
| 203 | Do you have abortion history? | Yes-----------------------------------1  No -------------------------------------2 |  |
| 204 | Do you want to conceive in the near future? | Yes-------------------------------------1  No --------------------------------------2 |  |
| 205 | If question-104-yes,  At what time you plan? | Within two years--------------------1  After two years-----------------------2 |  |

**1.3 Contraceptive related issues**

| **No** | **Questions** | **alternatives** | **skip** |
| --- | --- | --- | --- |
| 301 | Have you ever used any contraceptive prior to Implanon? | Yes---------------------------------------1  No ----------------------------------------2 |  |
| 302 | If question- 301-yes,  Which method have you used? | OCP --------------------------------------1  Injectable---------------------------------2  IUCD -------------------------------------3  Implants ----------------------------------4  Other (specify­)--------------------------5 |  |
| 303 | From where you did get Implanon? | Hospital -------------------------------1  Health center-----------------------------2  Health post ------------------------------3  Family guidance-------------------------4  Other(specify)-----------------------------5 |  |
| 304 | Did you get counseling service about benefit implanon? | Yes ----------------------------------------1  No -----------------------------------------2 |  |
| 305 | Did you get counseling, about possible side effect of implanon? | Yes ----------------------------------------1  No ------------------------------------------2 |  |
| 306 | Who choose Implanon? | My own ---------------------------1  My husband-------------------------2  Health Professional ----------------3  HEW----------------------------------4  Shared choice-----------------------5  Neighbors----------------------------6  Other(specify)------------------------7 |  |
| 307 | Do you have appointment for follow up? | Yes-------------------------------------------1  No --------------------------------------------2 |  |
| 308 | Removal due to side-effect? | Yes-----------------------------------------1  No-------------------------------------------2 |  |
| 309 | If question-308-Yes, which side effect you faced? | Menstrual Irregularity-------------------1  Weight gain -------------------------------2  Unusual headache----------------------3  Insertion of arm pain-------------------4  Difficult to work---------------------------5  Other(specify)-----------------------------6 |  |
| 310 | If question-308-No,  What are the none side effect removal reasons? | Husband objection----------------------1  No husband ------------------------------2  Husband go abroad---------------------3  Desire for pregnancy--------------------4  Other(specify)-----------------------------5 |  |
| 311 | Date of insertion of Implanon | ----------------------------------------------A |  |
| 312 | Date of removal | ----------------------------------------------B |  |
| 313 | Duration of Implanon utilization | =A-B--------------------------------(years) |  |

Thank you so much
